# Setting up a primary eye care teleconsultation service

**Published:** 2022-06-07

**Authors:** Padmaja Kumari Rani, Anthony Vipin Das

**Affiliations:** 1 Network Associate Director: Teleophthalmology, LV Prasad Eye Institute, Hyderabad, India.; 2 Department of eyeSmart EMR & AEye: LV Prasad Eye Institute, Hyderabad, India.

**Advances in information and communication technology have made it possible to set up teleconsultation services that can improve primary eye care.**

**Figure 1:**
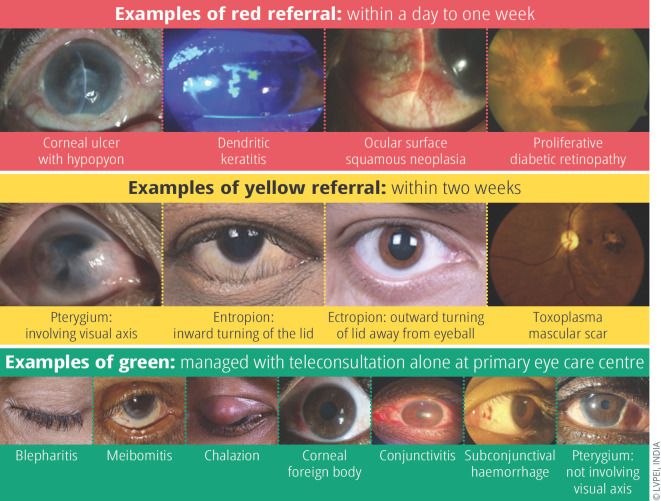
The referral and tracking system used in LV Prasad primary eye care vision centres.

**Cloud computing** is an umbrella term that includes storing data on a distributed network of servers that are connected via the internet, and using applications or apps that are hosted on this distributed network, which is also known as **‘the cloud’**.

**Synchronising data** (or syncing data) involves the background upload and download of data across a cloud service so that the most up-to-date data are available to everyone who is authorised to have access.

Advances in information and communications technology (ICT) have enabled us to scale up digital health solutions around the world. Medical teleconsultation has come a long way from the early explorations with television and telephone^1^ to the present use of smartphones and smart devices.

Today, primary eye care delivery can be greatly improved through ICT, enabling eye care staff at community or primary eye centres to be in direct contact with clinicians at tertiary centres and teaching hospitals. It is even possible for patients to speak directly with clinicians via online videoconferencing solutions.

Here are the key considerations when setting up a comprehensive primary eye care teleconsultation service.

## 
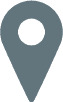
 Location

The location of the primary eye care centre or eye clinic (either a standalone facility or as part of a primary health centre) is crucial: it should ideally be near enough so that patients can afford to travel to the base hospital that provides higher medical and surgical care to patients.

## 
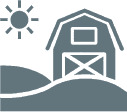
 Infrastructure

The primary eye care centre must have the space needed to carry out distance visual acuity tests (at a distance of 6 m) and near vision tests. The room should be well lit to allow staff members to carry out eye examinations.

The amount of space needed for the teleophthalmology command centre at the base hospital will depend on the number of teleconsultations per day and how many staff members have to work at the same time. The command centre would be staffed by ophthalmologists who could either work full time or allocate time for teleconsultations as a part of their weekly schedule.

## 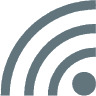 Connectivity

Reliable internet connectivity is crucial for seamless and smooth teleconsultation. If possible, a dedicated internet line with a Wi-Fi router should be installed in the primary eye care centre. Alternatively, a smart tablet with a 2G/3G/4G SIM card can be used to synchronise (sync) clinical information and teleconsultation requests through **cloud computing**.^2^ Where internet is erratic, digital apps can be used to store clinical information and data, which can be synced to the cloud when the internet connection is working. Another way to sync information to the cloud is to create an internet hotspot using a smartphone. In challenging environments, all options must be explored, and a hybrid model can be used to sync data from remote eye care centres to the cloud.

When patients are unable to come to the primary health centre, as for example during a pandemic, free video platforms such as Skype, WhatsApp, Google Meet, Microsoft Teams, and Zoom offer patients an easy way to communicate with the primary care provider.

## 
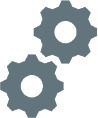
 Equipment

Visual acuity can be tested using a Snellen chart, digital screens, or through apps such as Peek Acuity and Smart Optometry.^3^

A basic set of equipment (trial lens set, slit lamp, and intraocular pressure measurement device) is necessary so that refractive errors and anterior segment disorders can be detected. A non-mydriatic fundus camera, if available, can be used to capture posterior segment pictures. Digital applications (apps) for teleconsultation can be installed on computers or smart tablets that run on Android or iOS.^4^ Alternatively, a Google Form (or similar) can be designed for use by primary eye care personnel to enter clinical data, which can then be accessed by the ophthalmologist.^5^

Clinical data and media captured by digital devices (cameras/scanners) must be shared in standard formats such as JPEG and MP4. The system must ensure secure transmission of data, ensuring patient confidentiality through encryption and password protection, and there must be defined levels of access for the care providers. Cloud service providers such as Amazon Web Service (AWS), Google Cloud, and Microsoft Azure can be used to integrate the teleconsultation system.

Ophthalmologists at the command centre can access teleconsultation requests on computer monitors and provide expert opinion within a time frame that is acceptable to the patient. A 3D-printed attachment holding a smart device to the slit lamp eyepiece^6^ can be used to live stream information during eye examination. A universal smartphone attachment can also be used to obtain better quality anterior segment pictures/videos for transmission to the command centre.^7^

## 
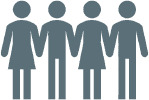
 Human resources

The primary eye care centre should ideally have a primary eye care provider trained in the basics of ocular anatomy, physiology, and pharmacology, who is able to identify common anterior and posterior segment eye conditions in patients presenting to the primary eye care centres.^8^

## 
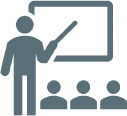
 Training

There should be regular training for primary eye care providers to update them with the latest clinical information, image capturing skills, and referral guidelines. There should also be regular certification programmes in teleconsulting, and anterior and posterior segment image capture. In India, the Ministry of Health and Family Welfare mandates that registered medical practitioners undergo an online course before practising teleconsultation.^9^

## 
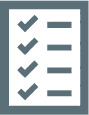
 Audit and monitoring

An easy way to monitor and support primary eye care providers across centres is through chat groups on free messaging and video calling apps, such as Telegram, Signal, Skype, Slack, and WhatsApp. Such a group can be used to quickly resolve technical issues and user queries.

Image quality is an essential factor in efficient teleconsultation. Primary eye care providers can be encouraged to post a ‘picture of the day’ in their chat groups to improve their imaging skills. Regular assessments can be instituted through weekly or monthly audit meetings, virtually or in person.

## 
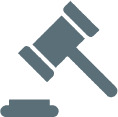
 Legal considerations

Government guidelines, as applicable, should be followed on the handling of patient-related health care data. Secure data transmission through encrypted channels and robust access protocols will protect data privacy and minimise the risk of data breaches. Legally acceptable patient consent must be obtained prior to teleconsultation and saved for future reference.

## 
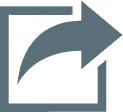
 Learning and improvement

Teleophthalmology referral guidelines can help to optimise the time of the treating ophthalmologist.^10^ Structured flow charts listing ocular conditions that can be referred for teleconsultation can be used in primary eye care centres. The teleophthalmology record system tracks referrals from primary to secondary/tertiary levels of care through a colour-coded system (Figure 1).

## 
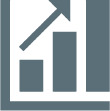
 Analytics

Analytics can give us better insight into the progress of the teleconsultation service. Data on patient demographics, clinical condition, triage categorisation, turnaround time, advice requested, and advice given must be analysed periodically. The graphical representation can be done on Microsoft Excel or visualised interactively using a platform such as Tableau or Power BI.

Finally, it is important to have the will to set up a teleconsultation service, especially considering the challenges that rural geography brings. However, the availability today of simple, free digital tools can minimise the cost of setting up such a service. Teleconsultation services offer a ray of hope to patients who may not otherwise have a chance to access quality eye care services.
